# Phosphate availability modulates elemental homeostasis in rainbow trout hepatocytes: compositional ionomics illuminates system-wide adjustments

**DOI:** 10.1093/mtomcs/mfag022

**Published:** 2026-06-12

**Authors:** Punidan D Jeyasingh, Nicholas Hrdlicka, Kristina D Baker, Ryan E Sherman, Matteo Minghetti

**Affiliations:** Department of Biology, Oklahoma State University, 501 Life Sciences West, Stillwater, OK 74078, Unites States; Department of Biology, Oklahoma State University, 501 Life Sciences West, Stillwater, OK 74078, Unites States; Department of Biology, Oklahoma State University, 501 Life Sciences West, Stillwater, OK 74078, Unites States; Department of Biology, Oklahoma State University, 501 Life Sciences West, Stillwater, OK 74078, Unites States; Department of Biology, Oklahoma State University, 501 Life Sciences West, Stillwater, OK 74078, Unites States

## Abstract

Phosphorus (P) is central to biology, yet it remains unclear whether P limitation acts as an isolated nutrient constraint or as a perturbation to a dynamical elemental network. We tested this using the rainbow trout liver cell line RTL-W1 by manipulating phosphorus supply (0%, 10%, and 100% of normal supply; P0, P10, and P100) and quantifying proliferation, protein content, metabolic activity, membrane integrity, and multielement composition. Phosphorus supply significantly altered cell proliferation and protein accumulation, with higher P supporting greater growth. Metabolic activity was affected by P supply, whereas membrane integrity remained largely stable, indicating altered allocation rather than generalized cellular damage. Compositional ionomic analysis revealed that phosphorus perturbation restructured the multielement network. At Day 3, treatment effects were strongest for P, K, and S. By Day 6, additional elements, including Sr and Mn, exhibited coordinated shifts relative to the P100 reference. Compositional data analysis showed that elemental imbalances shifted through time, consistent with flux rebalancing in an open system rather than static homeostasis. If phosphorus limitation were mechanistically independent, multielement composition would remain stable aside from P itself. Instead, phosphorus perturbation induced coordinated shifts across the ionome, consistent with rebalancing in open material systems.

## Graphical Abstract

**Graphical Abstract:**
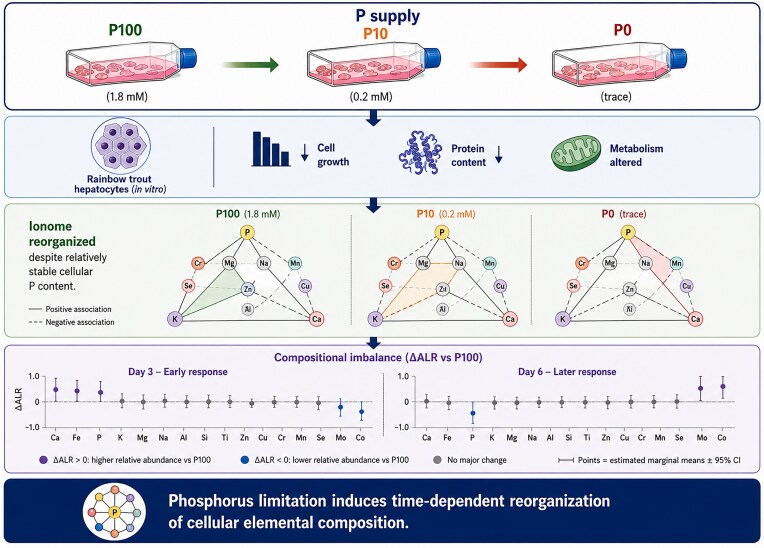
Phosphorus perturbation reorganized elemental homeostasis beyond phosphorus itself, revealing time-dependent rebalancing of a coupled cellular ionome.

## Introduction

Phosphorus (P) is central to cellular function because it is embedded in ATP, nucleic acids, phospholipids, and ribosomal RNA. Its biochemical indispensability has long been recognized [1] and its ecological importance is reflected in global concerns over P cycling and limitation [2, 3]. At the cellular scale, phosphorus availability is tightly linked to growth because rapidly dividing cells require increased ribosomal RNA to sustain protein synthesis, the basis of the Growth Rate Hypothesis [4].

Phosphorus limitation is not only a cellular constraint but a global sustainability concern. Disruption of the biogeochemical phosphorus cycle has been identified as one of the transgressed planetary boundaries, reflecting both finite geological reserves and large-scale anthropogenic mobilization [5]. In aquaculture and terrestrial animal production, phosphorus represents a valuable yet inefficiently utilized input. Because dietary P bioavailability is often low, supplementation is required, but substantial fractions are excreted, contributing to eutrophication and downstream water quality impairment [3, 6]. Understanding how phosphate availability reorganizes cellular metal and nutrient allocation therefore has implications extending from hepatocyte physiology to nutrient management at ecosystem scales.

While stoichiometric theory has elegantly linked nutrient supply to organismal growth and ecosystem dynamics [7, 8], most empirical work has focused on C:N:P ratios. Yet cells are not defined by three elements alone. Less is known about how phosphorus perturbation restructures the broader elemental network [9]. Essential metals such as Fe, Zn, Cu, and Mn function as enzyme cofactors and structural components [10, 11], and their cellular abundance is governed by coordinated transport and metabolic flux [12, 13]. Thus, altering phosphorus supply may propagate beyond P itself through coupled biochemical and transport networks.

As outlined in Box 1, cells are open, far-from-equilibrium systems sustained by continuous elemental fluxes rather than static quotas. In this framework, phosphorus is not merely a structural component but a regulator of energetic and biosynthetic throughput. Because ATP turnover, ribosome production, membrane synthesis, and metal-dependent catalysis are energetically and materially coupled, perturbing phosphorus supply is expected to reorganize multielement flux balances. Elemental composition at any moment therefore reflects the transient outcome of dynamic exchange, allocation, and redistribution within a constrained but open system. Ionomics provides a framework for quantifying this multielement structure [14, 15]. When analyzed compositionally, elemental concentrations reflect redistribution within a closed measurement system rather than independent variation [16].

**Box 1. RTL-W1 cells as dynamical material systems operating far from equilibrium**.

RTL-W1 hepatocytes are open chemical systems maintained far from thermodynamic equilibrium by continuous exchange of matter with the surrounding medium. At any time *t*, cellular elemental mass ${x_i}( t )$for element *i*changes according to the balance of fluxes:

\begin{eqnarray*}
\frac{{d{x_i}}}{{dt}} = J_i^{in} - J_i^{out} + J_i^{int}
\end{eqnarray*}

where $J_i^{in}$represents uptake from the medium, $J_i^{out}$represents efflux or excretion, and $J_i^{int}$represents internal transformation (e.g. incorporation into macromolecules, storage, or release from complexes).

Because cells grow, total elemental mass is not constant. Defining the measured elemental pool as

\begin{eqnarray*}
{F_v}\left( t \right) = \mathop \sum \limits_{i = 1}^D {x_i}\left( t \right)
\end{eqnarray*}

changes in composition necessarily reflect coordinated dynamics:

\begin{eqnarray*}
\frac{d}{{dt}}{\mathrm{ln}}\left( {\frac{{{x_i}}}{{{F_v}}}} \right) = \frac{1}{{{x_i}}}\frac{{d{x_i}}}{{dt}} - \frac{1}{{{F_v}}}\frac{{d{F_v}}}{{dt}}
\end{eqnarray*}

Thus, a perturbation to one elemental flux (e.g. reduced external phosphorus) alters both numerator and denominator dynamics. Even if absolute ${x_P}\,\,$stabilizes over time, shifts in uptake, growth rate, or allocation of other elements necessarily redistribute the entire elemental network.

Reduced phosphorus supply in this study induced an early compositional imbalance, followed by attenuation of the P signal and secondary redistribution into other elements. This temporal pattern is consistent with a far-from-equilibrium system that mitigates flux imbalance not by isolating a single element, but by coordinated rebalancing across the elemental pool. Importantly, because composition is inherently relational, no element can change independently. The ionome therefore provides a system-level diagnostic of how flux perturbations propagate through the cellular material network.

Here, using the rainbow trout liver cell line RTL-W1 [17], we experimentally varied phosphorus supply (i.e. normal—P100, 10% of normal—P10, and no P added—P0) and quantified cellular growth, protein content, metabolic activity, and multielement composition. The RTL-W1 cells were chosen for the central role of the liver in elemental homeostasis and metabolism [12]. We tested three hypotheses: (i) phosphorus supply alters growth and physiology (metabolic rate and protein content); (ii) phosphorus perturbation restructures the ionome as a coupled multielement system; and (iii) temporal shifts in elemental composition reflect far-from-equilibrium flux rebalancing rather than static homeostasis. If phosphorus acted only as a limiting nutrient in isolation, then only P would change. If instead cells behave as dynamical material systems (Box 1), multielement redistribution should occur and change directionally through time.

## Material and methods

### Experimental design and preparation of culture media

Synthetic exposure media with varying phosphorus concentration were prepared by modifying the composition of Leibovitz’s L-15 medium (Thermo Scientific, Waltham, USA) which is normally used to culture fish cells. Phosphorus concentration was manipulated by varying the concentration of sodium phosphate dibasic (Na_2_HPO_4_) in the media (0–1.8 mM of phosphate). The media were called P100, P10, and P0 to indicate the % or P relative to the Leibovitz’ L-15 commercial medium. Salts, sugars, amino acids, and vitamins concentration of P-modified media are identical to that of the commercial L-15 medium (Table 1). Salts and sugars were purchased from Sigma-Aldrich, vitamins and amino acids from Gibco, Thermo Fisher Scientific. All media contained 5% fetal bovine serum (FBS, Sigma-Aldrich, USA). To maintain a similar ionic strength and pH across all media, the phosphate concentration was substituted using HEPES (Sigma-Aldrich, St. Louis, MO, USA). The osmolality and the pH of each medium were measured using the Vapro® Vapor Pressure Osmometer (Model 5600, ELItech group, South Logan, UT, USA) and SI Analytics pH meter (SI Analytics, College Station, TX, USA), respectively. Essential trace metals (Cu, Fe, Zn) were largely present in the FBS [18], and thus did not vary among media with varying P concentrations (Table 1).

**Table 1:** Chemical composition and characteristics of cell culture media varying in phosphorus concentration

Salts and other components (mM)	L15 complete	P100	P10	P0
Cl	146.7	144.5	144.7	144.52
Ca	1.3	1.3	1.3	1.26
Mg	1.8	1.8	1.8	1.8
Na	144.2	145.6	140.4	140
K	5.8	5.8	5.8	5.77
PO_4_	1.8	1.8	0.2	0
SO_4_	0.8	0.9	0.8	0.81
HEPES	−	−	14.4	16
Pyruvate	5	5	5	5
Amino acid mix	26.2	26.2	26.2	26.2
Vitamin mix	2.1	2.1	2.1	2.1
Galactose	5	5	5	5
[P] from 5% FBS	0.002	0.002	0.002	0.002
Ionic strength (mmol/kg)	150.8	150.6	156.8	157.4
Osmolality (mmol/kg)	326.7	321.5	300.5	297
pH	7.2	7.25	7.05	7.01

### *In vitro* cell culture

RTL-W1 cells were kindly donated by Professor Kristin Schirmer (Eawag, Switzerland). Cells were cultured as previously described [17]. Briefly, cells were cultured in the commercial cell culture medium Leibovitz’ L-15 (Thermo Fisher Scientific, Waltham, USA) supplemented with 5% FBS (Sigma, USA) and 1% penicillin‐streptomycin (10 000 units/mL penicillin and 10 000 μg/mL of streptomycin; Sigma-Aldrich, St. Louis, USA) in 75 cm^2^ flasks (Greiner Bio-One, US) at 19°C and split into two flasks once they reached ~80%–90% confluency. For cell splitting, confluent flasks were washed twice with Versene (Thermo Fisher Scientific, Waltham, USA) and detached using 0.25% trypsin in phosphate buffer (Thermo Fisher Scientific, Waltham, MA, USA). When seeding cells for exposure experiments, cells were counted using an automated cell counter (Countess II automated cell counter, Thermo Fisher Scientific, Waltham, USA), and seeded at a concentration appropriate for the specific experiment (see below). Cells used in exposure experiments ranged from passage P62 to P74. Each experiment was conducted with cells of the same passage, while replicate trials used different passages.

### Cellular assays: metabolic activity, membrane integrity, and cell proliferation

Cells were seeded at 26 000/cm^2^ in 24-well plates containing modified L-15 media (Table 1).

At each time point (i.e. 1–8 days), the metabolic activity was measured using alamarBlue® (AB; Invitrogen, Eugene, OR, USA). Alamar Blue is a commercial preparation of the dye resazurin, which enters the cells and is reduced to resorufin by mitochondrial, microsomal, and cytosolic oxidoreductases and can be used as a marker of cell metabolic activity [19]. 5-carboxylfluorescein diacetate acetoxymethyl ester (CFDA-AM) is an esterase substrate that can enter into living cells as a nonfluorescent form and can be converted into a fluorescent product by different nonspecific cytoplasmic esterases. It can be used as an indicator of cell membrane integrity which is necessary to retain its fluorescent product [20].

To perform the assay, exposure medium was aspirated and cells were exposed to a solution of AB (5% v/v) and CFDA-AM (4 μM) both dissolved in the respective exposure medium. Exactly after 30 min, fluorescence was measured using the Cytation 5 plate reader (BioTek, USA) at excitation/emission wavelengths of 530/595 nm for AB, 485/530 nm for CFDA-AM. Following the alamarBlue assay, cell proliferation was measured on the same cells by staining the cell nuclei using NucBlue™ Live ReadyProbes™ Reagent (Hoechst 33342; Thermo Fisher Scientific, Waltham, USA) following manufacturer’s instructions. At each time point (i.e. 1–8 days), the cell nuclei were stained by adding one drop of NucBlue per well and incubating the cells for 15 minutes. Images were taken using the 20X objective and the DAPI filter (excitation/emission 377/447) using the Cytation 5 imaging system (BioTek, USA). Cell nuclei were counted automatically using the Gen5 software (BioTek, USA; Figure S1). In each experiment, we collected one field of view (394 × 291 μm; see Fig. S1) per well and collected images from four separate wells (i.e. technical replicates) for each treatment. This experiment was repeated three times in separate trials.

### Elemental and protein quantification

For ionomic profiling, RTL-W1 cells were seeded at the same density as in metabolic and proliferation assays (26 000 cells/cm²), but in six-well plates to obtain sufficient biomass. At each time point, protein quota was measured from the same cultures used for ionomic analysis. Cells were washed twice with phosphate-free physiological buffer (L-15/P-free; Table 1) and lysed in 50 mM NaOH for two hours. Ten percent of the lysate was used for protein quantification via a Lowry assay [21]. The remaining lysate was desiccated (Concentrator Plus, Eppendorf, USA), digested overnight in 0.8 mL 69% HNO₃ and 0.2 mL H₂O₂, diluted 10 × with ultrapure water (16–18 MΩ·cm^−1^; Thermo Fisher Scientific), and analyzed using ICP-OES (Thermo Scientific iCAP 7400). ICP–OES measurements were calibrated using multielement external standards and an in-line yttrium internal standard to correct for drift and matrix effects. Elements with concentrations at or below blank levels were considered below detection limits and excluded. To ensure robust and comparable quantification across all samples, analyses were restricted to elements consistently above detection limits, resulting in a dataset of 14 elements (Ca, K, Mg, P, S, Cu, Zn, Fe, Mn, Ni, Cr, Co, Sr, Mo). While higher-sensitivity techniques such as ICP–MS can resolve additional trace elements, inclusion of elements with inconsistent detectability would introduce noise and reduce comparability across samples. Because our inferences are based on coordinated, treatment-dependent shifts among reliably quantified elements, the system-level patterns reported here do not depend on trace elements near detection limits. Baseline (Day 0) and FBS medium samples were included for descriptive comparison but excluded from primary treatment contrasts.

### Construction of compositional coordinates

As introduced in Box 1, because elemental measurements represent parts of a multivariate whole, ionomic data were analyzed within a compositional data framework. For each sample, we defined the filling value (Fv) as the sum of all reliably quantified elements within that sample:


\begin{eqnarray*}
{\mathrm{Fv}} = {\Sigma _{\mathrm{i}}}{{\mathrm{x}}_{\mathrm{i}}}
\end{eqnarray*}

where x_i_ is the measured concentration of element i. Duplicate columns were removed prior to analysis. Elements with unstable or missing values were excluded from Fv calculation where necessary. Fourteen elements, Ca, K, Mg, P, S, Cu, Zn, Fe, Mn, Ni, Cr, Co, Sr, and Mo, were reliably measured in this study.

Elemental composition was expressed as a form of additive log-ratios (ALRs), defined as:


\begin{eqnarray*}
{\mathrm{AL}}{{\mathrm{R}}_{\mathrm{i}}} = \ln \left( {{{\mathrm{x}}_{\mathrm{i}}}/{\mathrm{Fv}}} \right)
\end{eqnarray*}

This transformation places all elements in a common, dimensionless coordinate system and preserves subcompositional coherence [22]. Following best practices, analyses were repeated using centered log-ratios to confirm that qualitative inference was unchanged.

Because ionomic composition changes over time in culture, primary treatment effects were evaluated within day for cell samples (Day 3 and Day 6 analyzed separately). For each day, overall compositional differences among phosphorus treatments were tested using multivariate analysis of variance (MANOVA) with the full ALR matrix as the response: [ALR₁, ALR₂, …, ALR_D_] ∼ Treatment. Pillai’s trace was used to evaluate multivariate significance. Following significant MANOVA results, element-specific linear models were fitted: ALR_i_ ∼ Treatment.

## Results

**Figure 1:**
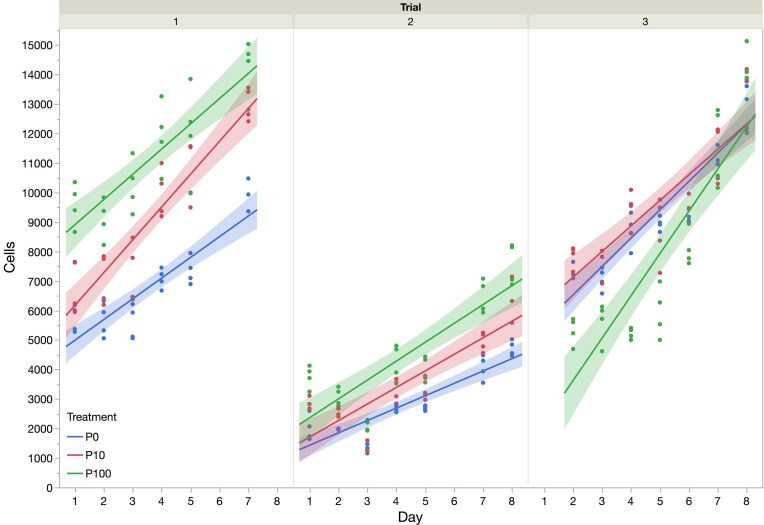
Cell number (cells well⁻¹) plotted against day of culture for P0 (trace P), P10 (0.2 mM P), and P100 (1.8 mM P). Points represent individual wells (*n* = 4 per treatment per day). Lines show linear regressions fit separately within each trial using the model Cells ∼ Day × Treatment; shaded regions indicate 95% confidence intervals of fitted lines. Growth rate (slope) differed among treatments (Table S1). In Trial 1, P10 exhibited a significantly steeper slope than P0, whereas P100 did not differ from P0. In Trials 2 and 3, P100 exhibited significantly steeper slopes than P0, whereas P10 did not differ significantly from P0. Across trials, complete phosphate omission (P0) consistently reduced proliferation relative to at least one phosphate-supplemented treatment.

**Figure 2:**
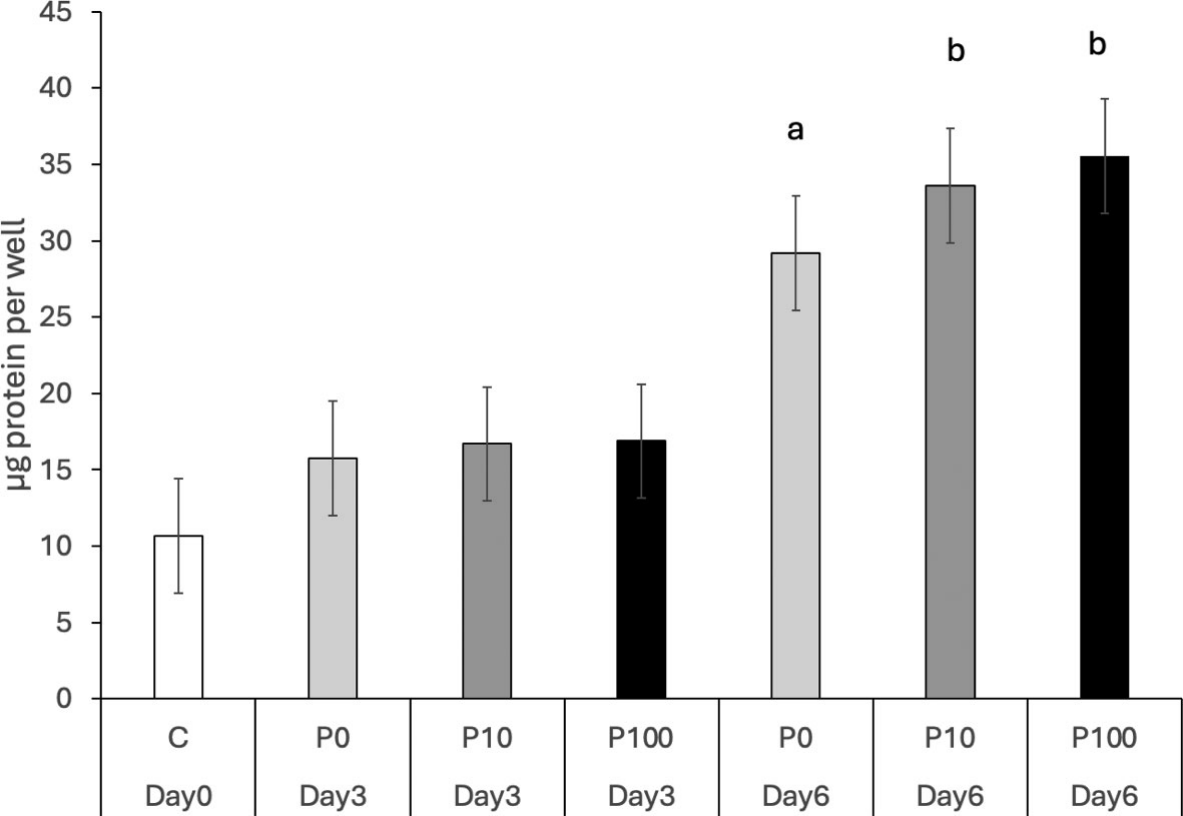
Protein content (µg protein per well) of RTL-W1 cells cultured under P0, P10, and P100 conditions. Points represent biological replicates; bars show means ± 95% confidence intervals. Phosphorus treatment did not affect protein content at Day 3 (*F*₂,₆ = 0.77, *P* = .503), but significantly affected protein at Day 6 (*F*₂,₆ = 9.48, *P* = .013). Bars bearing different letters are significantly different (one-way ANOVA followed by Tukey post hoc test, *P* < .05).

**Figure 3:**
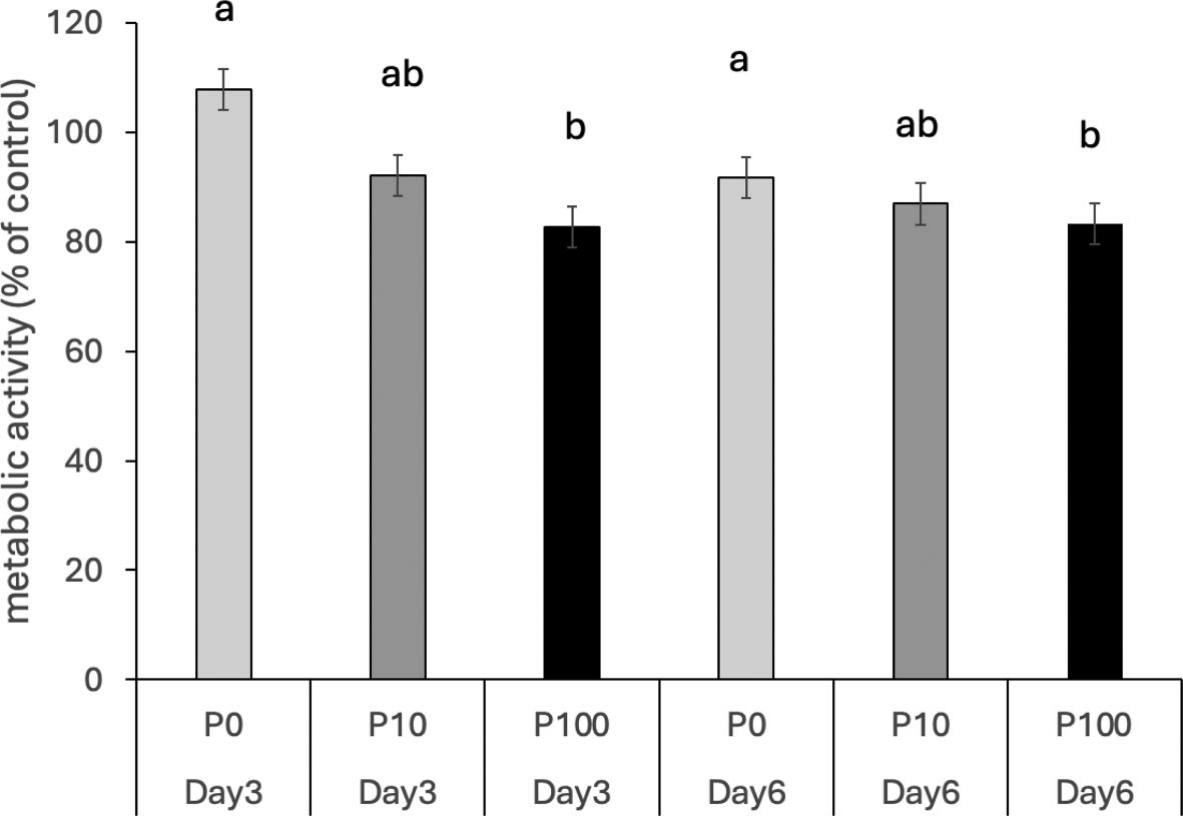
Effects of phosphorus supply on metabolic activity of RTL-W1 cells at Day 3 and Day 6. Bars represent means ± 1 SE pooled across trials. Metabolic activity (Alamar Blue reduction; % of control) was significantly affected by phosphorus treatment (*F*₂,₅₄ = 6.03, *P* = .0043). Tukey post hoc comparisons indicated that P0 exhibited higher metabolic activity than P100 (*P* = .005), whereas P10 did not differ significantly from either treatment (different letters indicate significant differences). See Fig. S1 for raw fluorescence data.

**Figure 4:**
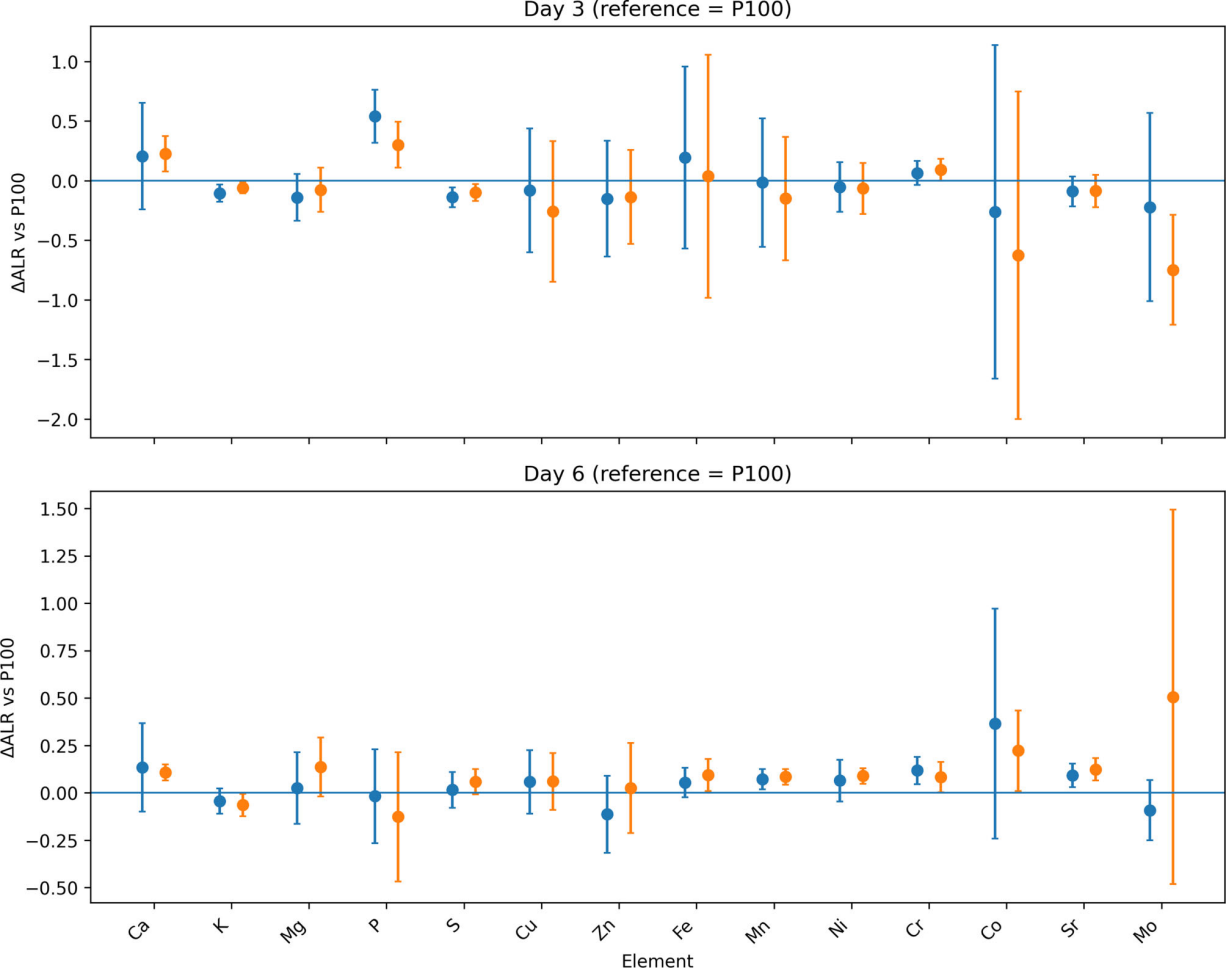
Element-specific compositional shifts are shown as additive log-ratio contrasts (ΔALR) for P0 (blue) and P10 (orange) relative to the P100 treatment within each day. Values represent estimated marginal means ± 95% confidence intervals from linear models fit separately for each element within day. The horizontal line at zero denotes no difference from P100. Positive values indicate that the element constitutes a larger fraction of the measured elemental pool (Fv) relative to P100, whereas negative values indicate relative depletion. Because data are expressed in log-ratio coordinates, all shifts reflect coordinated redistribution within a closed multielement system rather than independent changes in absolute concentration. Top panel: Day 3. Bottom panel: Day 6.

Phosphorus supply altered the growth and physiology of RTL-W1 cells as quantified by altered metabolic activity, protein quota, and ionome. Proliferation increased linearly with time in all three trials, and within each trial phosphorus treatment significantly affected growth, with P10 and P100 generally exceeding P0 across days (Fig. 1). Protein quota increased strongly through time; treatment differences were minimal at Day 3 but emerged by Day 6, when protein was lowest under P0 and higher under P10 and P100 (Fig. 2). Metabolic activity (alamarBlue) showed a significant phosphorus effect when pooled across trials, whereas membrane integrity (CFDA–AM) did not show a significant treatment effect (Fig. 3). In contrast to single-endpoint summaries, ionomics revealed that phosphorus supply significantly altered multivariate elemental composition at both Day 3 and Day 6, with an early P-centered imbalance that attenuated by Day 6 as other elements shifted (MANOVA; Fig. 4; Table S2).

### Cellular growth

To test whether phosphorus availability altered growth rate, we fitted a linear model within each trial including a Day × Treatment interaction. A significant interaction term indicates differences in slopes (cells·day⁻¹) among treatments.

In Trial 1, slopes differed significantly among treatments (Day × Treatment: *F*_2,66_ = 4.47, *P* = .015). Post hoc contrasts indicated that P10 exhibited a significantly steeper growth rate than P0, whereas P100 did not differ significantly from P0. In Trial 2, slope differences were not statistically significant (Day × Treatment: *F*_2,78_ = 2.70, *P* = .074), indicating parallel growth trajectories among treatments. In Trial 3, slopes again differed significantly among treatments (Day × Treatment: *F*_2,78_ = 6.92, *P* = .0017). Post hoc contrasts indicated that P100 exhibited a significantly steeper slope than P0, whereas P10 did not differ significantly from P0.

Across trials, complete phosphate omission (P0) reduced growth rate relative to at least one phosphate-supplemented treatment, although the relative ranking of P10 and P100 varied among trials.

### Protein quota

Protein content increased substantially from Day 0 to Day 6 across all treatments (Fig. 2). At Day 3, phosphorus treatment did not significantly affect protein content (one-way ANOVA: *F*₂,₆ = 0.77, *P* = .503). Mean (± SD) protein content at Day 3 was 16.88 ± 2.93 µg well⁻¹ (P100), 16.69 ± 0.36 µg well⁻¹ (P10), and 15.75 ± 0.88 µg well⁻¹ (P0).

At Day 6, phosphorus treatment significantly affected protein content (*F*₂,₆ = 9.48, *P* = .013). Mean protein content was highest in P100 (35.54 ± 2.75 µg well⁻¹) and P10 (33.60 ± 4.18 µg well⁻¹), and lower in P0 (29.18 ± 1.00 µg well⁻¹). Tukey post hoc comparisons indicated that both P100 and P10 exceeded P0 (*P* < .05), whereas P10 did not differ significantly from P100.

### Metabolic rate and membrane integrity

Metabolic activity, quantified as alamarBlue fluorescence reduction (AB; % of control), was significantly affected by phosphorus treatment when data were pooled across trials and analyzed with day included as a covariate (Fig. 3; Fig. S1; *F*₂,₅₄ = 6.03, *P* = .0043). Tukey post hoc comparisons indicated that P0 exhibited significantly higher metabolic activity than P100 (*P* = .005), whereas P10 did not differ significantly from either P0 or P100 (*P* > .23).

In contrast, membrane integrity, measured as CFDA-AM fluorescence (% of control), was not significantly affected by phosphorus treatment (Fig. S2; *F*₂,₅₄ = 0.78, *P* = .461). Thus, while phosphorus availability influenced metabolic performance, it did not produce consistent treatment effects on membrane stability.

### Ionomics

Phosphorus supply significantly altered multivariate elemental composition at both sampling times (MANOVA, Pillai’s trace, *P* < .05 for Day 3 and Day 6). To identify specific contributors to multivariate separation, we fit element-specific linear models within each day to diagnose element-specific treatment effects (Table S2). Within Day 3, treatment effects were strongest for P, with additional nominal effects for K and S. By Day 6, nominal treatment effects were observed for Sr, Cr, and Mn. For interpretability, treatment contrasts are therefore emphasized via ALR effect sizes relative to P100 with 95% confidence intervals (Fig. 4), which visualize coordinated shifts of the ionome within the measured elemental pool (Fv).

## Discussion

This study was designed as a test of how phosphorus supply restructures cellular performance and elemental composition in a vertebrate cell line. Rather than asking whether phosphorus affects growth in isolation, we asked whether phosphorus perturbation reorganizes growth, metabolism, and the ionome as a coupled system operating far from equilibrium. Our results provide evidence for systemic adjustments to P supply.

### Phosphorus limitation inhibits growth and alters metabolism without generalized cellular failure

Phosphorus supply significantly affected proliferation across all three trials (Fig. 1), altered protein quota by Day 6 (Fig. 2), and modified metabolic activity (Fig. 3). Thus, P limitation modulated growth and allocation rather than inducing wholesale cytotoxic collapse.

This pattern is consistent with classical phosphorus physiology in fish and other vertebrates, where P restriction reduces growth efficiency and protein deposition before overt tissue damage occurs [6, 8, 23, 24]. Phosphorus is central to ATP, nucleotides, and ribosomal RNA [1, 25], and limitation is therefore expected to constrain biosynthetic throughput before membrane stability fails. Moreover, P limitation might be compensated by inorganic phosphate reserves (PolyP) which are buffering initial P limitation at the cellular level [26]. The dissociation between metabolic activity and membrane integrity further indicates that cells reprogram energy allocation under P limitation rather than simply losing viability. Furthermore, the increase in cellular metabolic activity, recorded at Day 3, can be explained by a general stress response driven by the increased energy demands required to maintain homeostasis, manage damage, and trigger protective mechanisms as seen in fish exposed to osmotic stress [27, 28].

Importantly, growth responses were not identical across trials (Fig. 1). Initial cell density varied across the cell proliferation trials which might have contributed to the variation. Considering that we have used an automated cell counter, this difference could be attributed to the variation in passage number used in the different trials, which might have affected the cell attachment and proliferation [29]. Regardless, the direction of treatment separation was consistent: P100 and P10 generally exceeded P0 in proliferation and protein by Day 6. This repeatable directional response strengthens the interpretation that phosphorus availability constrains anabolic throughput rather than causing stochastic stress responses.

### Phosphorus restructures the ionome as a coupled system

The key result is not that cellular P changes; it is that multielement composition shifts in a coordinated manner (Fig. 4). MANOVA detected significant multivariate treatment effects within both Day 3 and Day 6, and element-specific ALR analyses identified significant or nominal shifts in P, K, S (Day 3) and Sr, Mn, Cr (Day 6) (Table S2). These changes are expressed in log-ratio space relative to the measured elemental pool (Fv), meaning they reflect redistribution within a closed multielement system rather than independent fluctuations in absolute concentration.

If phosphorus acted purely as an isolated limiting substrate, one would expect changes confined largely to P itself. Instead, P perturbation propagated across multiple elements, altering their proportional representation within the cellular ionome (Fig. 4). This pattern is consistent with the ionomics perspective that treats elemental composition as an integrated phenotype shaped by shared transporters, cofactors, and metabolic coupling [14, 15, 30].

Phosphorus metabolism is mechanistically linked to metal homeostasis. Ribosome production, ATP-dependent enzymes, and kinase networks impose coordinated demands on Mg, K, and transition metals [10–12]. The drop in K intracellular concentration is interesting as we could speculate that could be due to a drop in intracellular ATP (not measured), which in turns could result in a reduced Na/K-ATPase activity thus affecting cellular K. The observed decrease in intracellular K is consistent with reduced ATP-dependent Na/K-ATPase activity under P limitation [31]. Furthermore, growth dilution and allocation shifts are known to restructure elemental quotas in aquatic systems [32–34]. Thus, the multielement shifts observed here are expected under a network constraint model but not under a strictly Liebig-type independent-element model [35]. In short, phosphorus limitation reorganized the RTL ionome as a system.

### Time-dependent rebalancing reflects far-from-equilibrium flux adjustment

The temporal shift between Day 3 and Day 6 provides the strongest mechanistic signal. At Day 3, phosphorus imbalance (relative to P100) is pronounced in P0 and P10 (Fig. 4, top panel). By Day 6, P imbalance is damped, yet other elements, most strikingly Sr, and to a lesser extent Mn and Cr, shift directionally (Fig. 4, bottom panel). This pattern indicates not static homeostasis but dynamical rebalancing.

Living cells are open systems that maintain structure through continuous fluxes of matter and energy [36]. Under P perturbation, fluxes of ATP production, ribosome synthesis, ion transport, and protein turnover must be reallocated. Because ATP-consuming processes are hierarchically organized [37] and protein synthesis is energetically expensive [38, 39], shifts in P supply will cascade through other elemental demands. The observed damping of P imbalance alongside redistribution of Sr [40] and other elements is consistent with compensatory flux adjustment rather than restoration to an identical compositional steady state.

This interpretation aligns with multivariate imbalance theory applied in consumer systems [23]. However, here the framework is resolved at the cellular scale. The RTL system thus demonstrates that elemental rebalancing unfolds through time as cells adjust fluxes, not merely static pools. If phosphorus only mattered for “P-intensive” processes, then multielement redistribution would not appear, and it would not change directionally between Day 3 and Day 6. The data contradict that null expectation.

### Biochemical anchors and mechanistic plausibility

To our knowledge, there is no published transcriptome of phosphorus-limited RTL-W1 cells. However, dietary P restriction in rainbow trout induces coordinated transcriptional reprogramming, particularly in skeletal and mineralization pathways [6]. In low-P trout, RNA-seq revealed differential expression of genes central to bone mineralization and extracellular matrix regulation, implicating coordinated shifts in other biogenic elements such as calcium and magnesium and broader mineral homeostasis pathways [23]. These data demonstrate that phosphorus perturbation propagates beyond simple P handling into structural and regulatory networks. As discussed here, ionomic data is a way to quantitatively capture such systemic responses.

These systemic responses are qualitatively similar to other systems, phosphorus limitation consistently reorganizes translation, energy metabolism, and membrane processes. In *Daphnia*, P supply alters expression of ribosomal, metabolic, and stress-response genes [41, 42]. Plants and algae show similar genome-wide restructuring of central metabolism and membrane composition under phosphate deprivation [43, 44]. These responses converge on growth rate control, redox balance, and membrane remodeling. Such transcriptional shifts have predictable elemental consequences. Ribosomal RNA and ATP bind Mg²⁺, coupling P demand to Mg allocation [1, 25]. Protein synthesis is one of the most ATP-intensive cellular processes [37, 39], so altered growth under P limitation should restructure energetic and cofactor demand. Likewise, respiratory and redox enzymes depend on Fe-, Cu-, and Mn-containing metalloproteins [10, 11]. The redistribution of Mn, Fe, and Sr between Day 3 and Day 6 is therefore biochemically plausible as compensatory adjustment of metabolic and membrane-associated fluxes.

Although phosphate signaling, mineralization, translation, and redox regulation appear distinct, they share a common elemental substrate. As emphasized by R. J. P. Williams, life evolved by selecting elements whose coordination chemistry and redox properties match biological function [45, 46]. ATP binds Mg²⁺ because phosphate chemistry demands charge stabilization; redox enzymes use Fe, Cu, and Mn because their electrochemical potentials fall within biological windows. These are chemically constrained solutions, not arbitrary ones. Under this framework, phosphorus limitation perturbs a chemically coupled system. If ATP demand shifts, Mg coordination shifts. If translation slows, metal requirements change. If redox balance is adjusted, Fe-, Cu-, and Mn-dependent enzymes are redeployed. The multielement redistribution observed here is therefore consistent with conserved biochemical logic rather than independent element-by-element effects.

To our knowledge, there are no experiments measuring responses of human hepatocyte models (e.g. HepG2) to P supply. Studies leveraging such models have largely focused on pathway-specific or transcriptomic responses to metabolic stress [47], rather than the dynamical multielement composition measured here. Applying such an approach could help resolve how phosphorus availability propagates through the broader elemental network in human systems, providing a mechanistic bridge between systemic cellular metabolism and dynamical nutrient imbalance, illuminating novel remedial strategies such as nutrition-dependent drug metabolism [48].

### Scope, limitations, and conclusions

This study was designed as a controlled test of whether phosphorus supply alters only P-dependent processes or reorganizes a broader elemental network. By integrating growth, allocation, metabolic activity, and compositional ionomics within a single framework, we evaluated cellular performance and chemistry simultaneously. The convergence of functional responses (Figs 1 –3) with coordinated, time-dependent ionomic shifts (Fig. 4; Table S2) supports the network interpretation: phosphorus availability restructures multielement composition rather than affecting P alone.

Several limitations warrant consideration. We quantified a defined set of reliably measured elements, not the full elemental complement of the cell; thus, unmeasured components contribute to the true denominator. ICP-based measurements also carry standard analytical uncertainties, including matrix effects and detection limits for trace metals. However, analytical noise does not account for coordinated, treatment-dependent patterns that exceed instrument variation and recur across time. Because inference is based on coordinated shifts among consistently quantified elements, inclusion of other elements is not required to support the observed system-level patterns. Finally, RTL-W1 cells represent a simplified hepatic model and do not capture whole-organism feedbacks (including the influence of bone; [23], tissue partitioning, or dietary complexity. This limits extrapolation but strengthens systemic inference at the cellular scale. At the same time, the absence of organismal buffering allows direct resolution of cell-intrinsic elemental reallocation in response to phosphorus perturbation.

Measurement uncertainty or incomplete elemental coverage does not negate the underlying dynamical system. Cells remain open systems exchanging matter with their environment [36]. Altering phosphorus supply necessarily perturbs fluxes governing ATP production, protein synthesis, and metal-dependent enzymatic networks [10, 37]. The attenuation of P imbalance from Day 3 to Day 6, accompanied by redistribution of elements such as Sr and Mn (Fig. 4), is consistent with time-dependent flux rebalancing rather than static homeostasis.

We therefore conclude that phosphorus limitation should be examined not solely through P-specific pathways or C:N:P ratios, and their associated biochemical “chaperones”, but through the architecture of coupled elemental systems. The coordinated multielement shifts observed here clearly indicate a networked response consistent with far-from-equilibrium biological systems [36]. Compositional ionomics quantitatively links molecular biology and elemental biology.

## Supplementary Material

mfag022_Supplemental_Files

## Data Availability

The datasets generated and analyzed during this study are available as online supplement.
