# Supplementary material for: Phosphate availability modulates elemental homeostasis in rainbow trout hepatocytes: compositional ionomics illuminates system-wide adjustments
Source: Metallomics. 2026 Jun 12;18(1):mfag022. doi: 10.1093/mtomcs/mfag022 (PMC13321119; doi:10.1093/mtomcs/mfag022)

*Supplementary Table 1.* ANOVA summaries for phosphorus treatment (P0, P10, P100) effects on growth, protein, metabolic activity, and membrane integrity.

| Response Variable | Subset / Model | df  (model, error) | F | p |
| --- | --- | --- | --- | --- |
| **Cell proliferation** | **Trial 1** | **2, 66** | **124.71** | **<0.0001** |
|  | **Trial 2** | **2, 78** | **26.27** | **<0.0001** |
|  | **Trial 3** | **2, 78** | **17.36** | **<0.0001** |
| **Protein quota** | Day 3 | 2, 6 | 0.77 | 0.503 |
|  | **Day 6** | **2, 6** | **9.48** | **0.013** |
| **Metabolic activity (AB)** | **Days pooled (as covariate)** | **2, 54** | **6.03** | **0.0043** |
| **Membrane integrity (CFDA)** | Days pooled (as covariate) | 2, 54 | 0.78 | 0.461 |

*Supplementary Table 2:* Element-specific ALR ANOVAs within day. ALR = ln(element/Fv), analyzed separately for Day 3 and Day 6. Results of one-way ANOVA of Treatment (P0, P10, P100) within each day.

| Element | Day 3  F_2,6_ | Day 3  p | Day 6  F_2,6_ | Day 6  p |
| --- | --- | --- | --- | --- |
| Ca | 0.84 | 0.476 | 1.04 | 0.410 |
| Co | 0.52 | 0.620 | 0.95 | 0.439 |
| **Cr** | 1.43 | 0.310 | **5.69** | **0.041** |
| Cu | 0.35 | 0.720 | 0.45 | 0.656 |
| Fe | 0.11 | 0.896 | 2.76 | 0.141 |
| **K** | **5.97** | **0.037** | 2.75 | 0.142 |
| Mg | 1.17 | 0.374 | 1.65 | 0.268 |
| **Mn** | 0.24 | 0.793 | **5.40** | **0.046** |
| Mo | 2.58 | 0.156 | 1.22 | 0.360 |
| Ni | 0.17 | 0.851 | 1.96 | 0.222 |
| **P** | **11.85** | **0.008** | 0.41 | 0.682 |
| **S** | **6.13** | **0.036** | 0.86 | 0.468 |
| **Sr** | 1.55 | 0.286 | **8.87** | **0.016** |
| Zn | 0.27 | 0.769 | 1.08 | 0.398 |

*Supplementary Figure 1:* Mean ± SD (N=4) raw fluorescence units (F.U.) of alamarBlue. Bars bearing different lettering are statistically different (2- way ANOVA, Tukey as post hoc, P < 0.05).


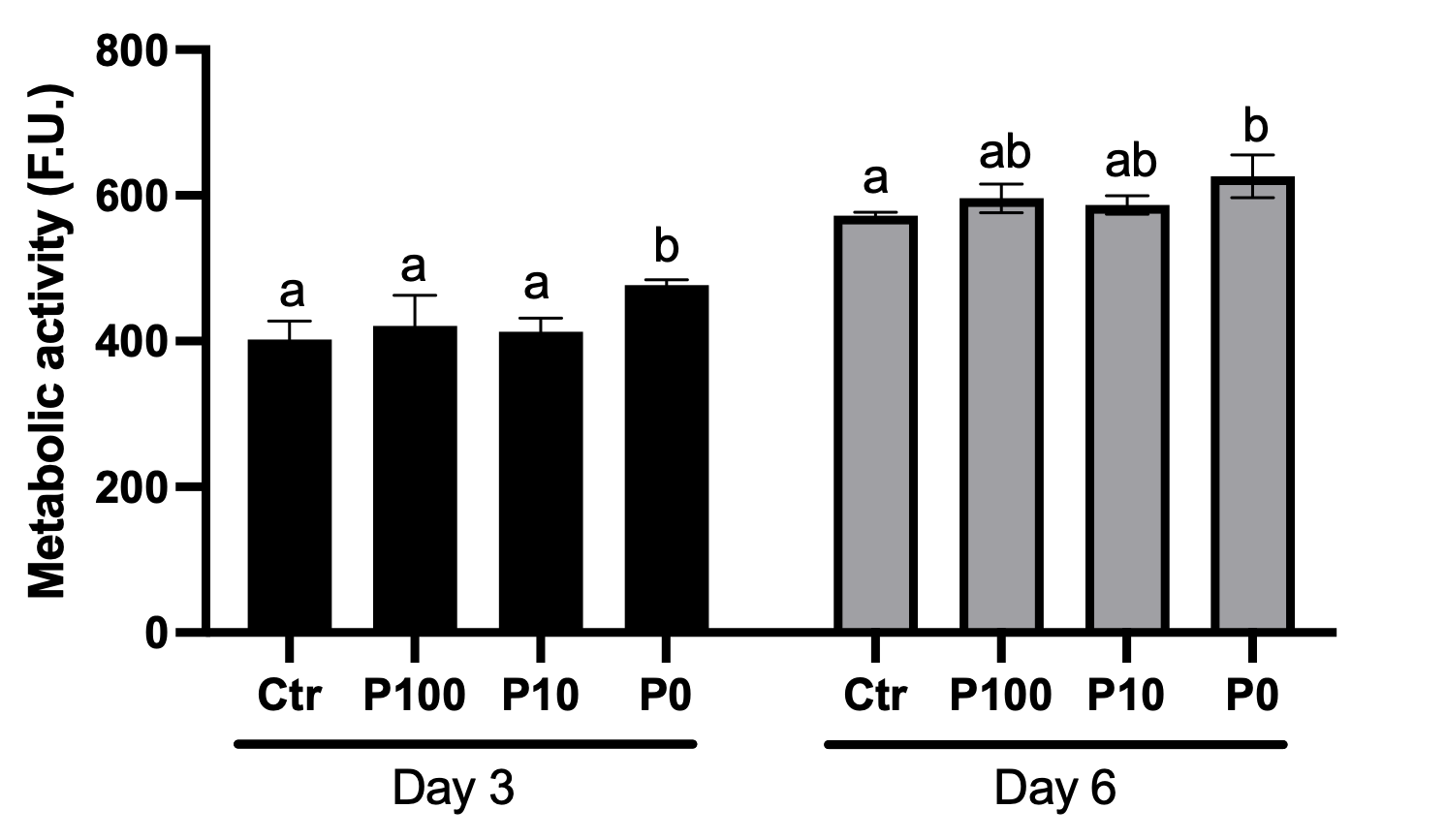


*Supplementary Figure 2:* Membrane integrity (**CFDA**) of RTL-W1 cells at Day 3 and Day 6 in each of the treatments. Bars represent means ± 1 SE pooled across trials. A) % of control, b) raw fluorescence units (F.U.) of **CFDA.**

a)


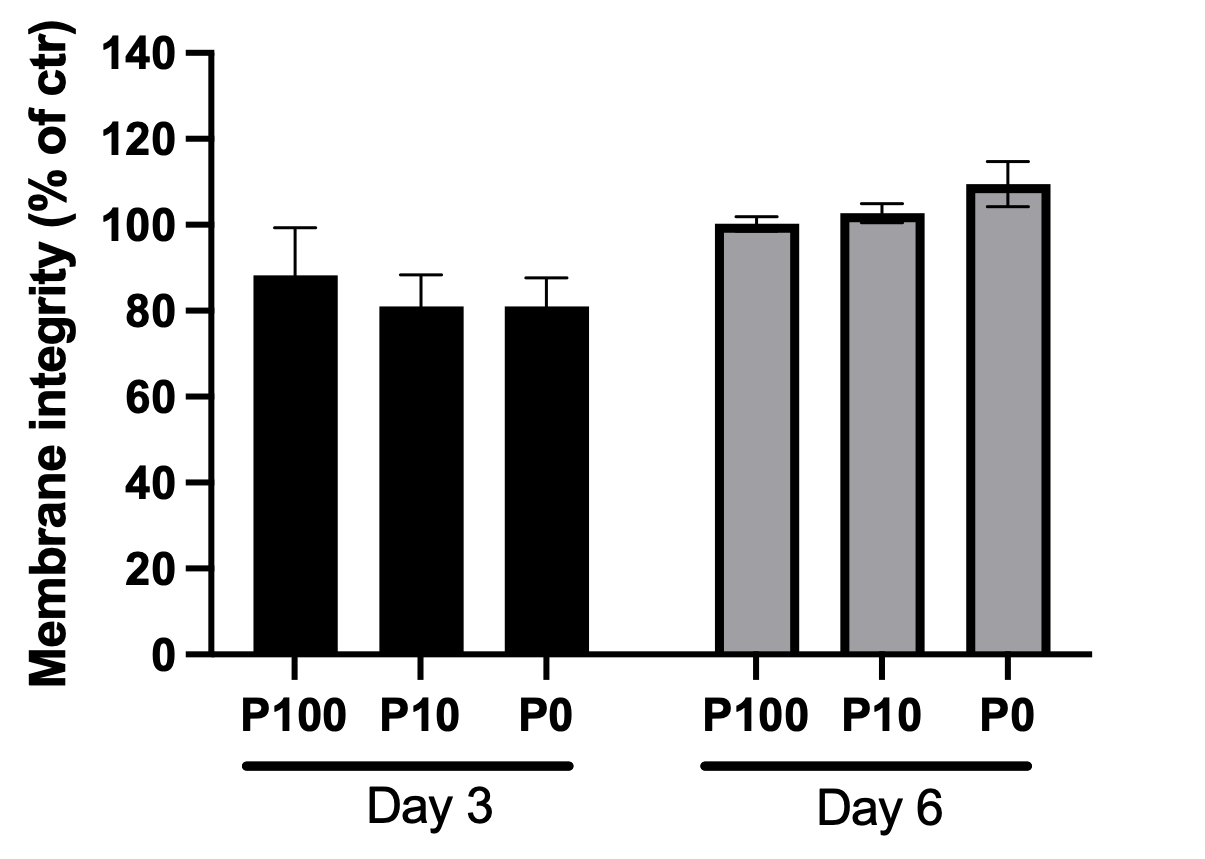


b)


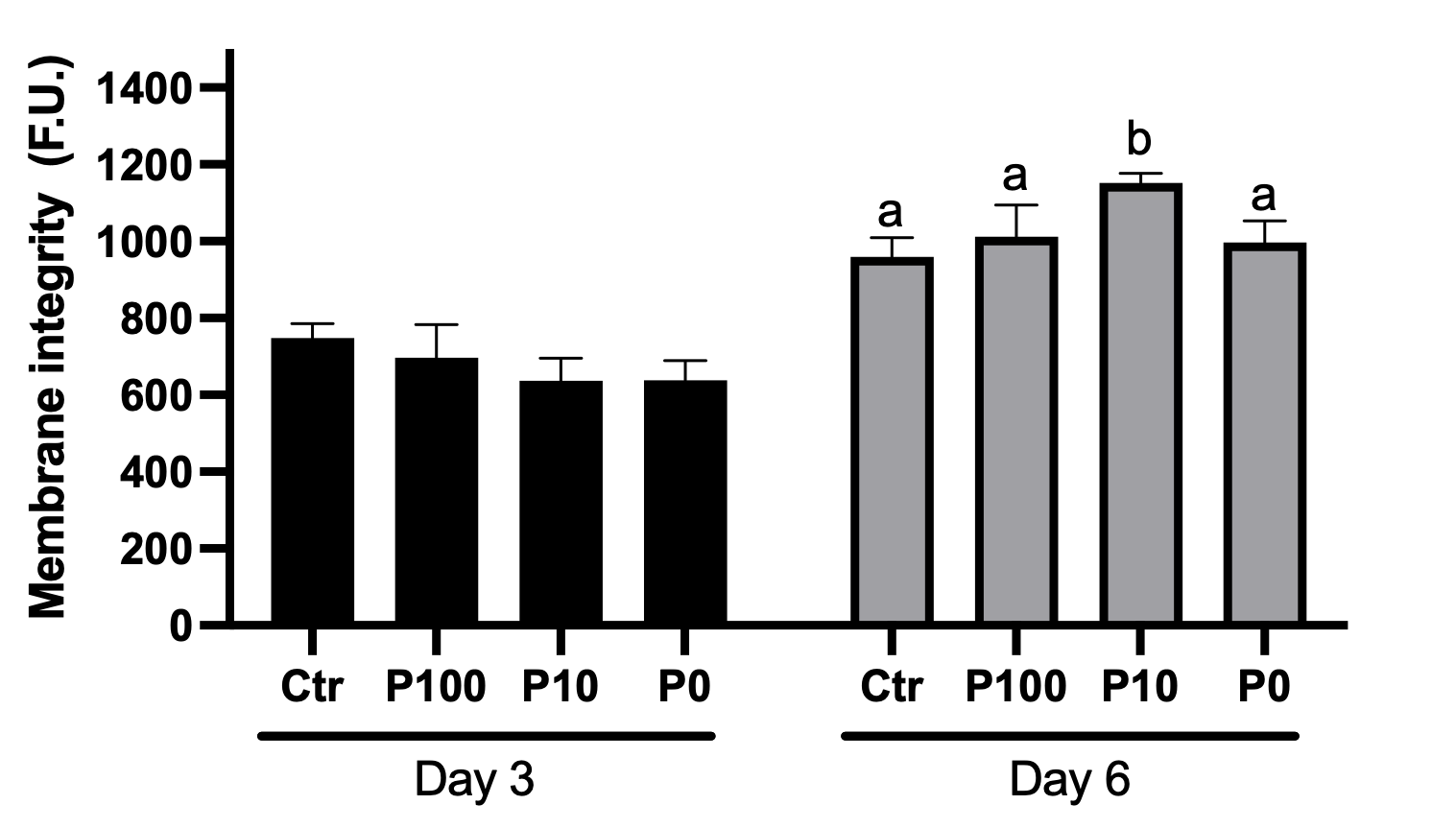

Supplement: mfag022_Supplemental_Files [file mfag022_supplemental_files.zip › Supplementary Materials - Tables and Figures.docx]
